# Enhanced reactivity of Li^+^@C_60_ toward thermal [2 + 2] cycloaddition by encapsulated Li^+^ Lewis acid

**DOI:** 10.3762/bjoc.20.58

**Published:** 2024-03-25

**Authors:** Hiroshi Ueno, Yu Yamazaki, Hiroshi Okada, Fuminori Misaizu, Ken Kokubo, Hidehiro Sakurai

**Affiliations:** 1 Creative Interdisciplinary Research Division, Frontier Research Institute for Interdisciplinary Sciences (FRIS), Tohoku University, 6-3 Aoba, Aramaki, Aoba-ku, Sendai 980-8578, Japan; 2 Department of Chemistry, Graduate School of Science, Tohoku University, 6-3 Aoba, Aramaki, Aoba-ku, Sendai 980-8578, Japan; 3 Division of Applied Chemistry, Graduate School of Engineering, Osaka University, 2-1 Yamadaoka, Suita, Osaka 565-0871, Japan

**Keywords:** electron transfer, fullerene, ion-endohedral fullerene, Lewis acid catalyst, thermal [2 + 2] cycloaddition

## Abstract

Lithium ion-endohedral fullerene (Li^+^@C_60_), a member of the burgeoning family of ion-endohedral fullerenes, holds substantial promise for diverse applications owing to its distinctive ionic properties. Despite the high demand for precise property tuning through chemical modification, there have been only a few reports detailing synthetic protocols for the derivatization of this novel material. In this study, we report the synthesis of Li^+^@C_60_ derivatives via the thermal [2 + 2] cycloaddition reaction of styrene derivatives, achieving significantly higher yields of monofunctionalized Li^+^@C_60_ compared to previously reported reactions. Furthermore, by combining experimental and theoretical approaches, we clarified the range of applicable substrates for the thermal [2 + 2] cycloaddition of Li^+^@C_60_, highlighting the expanded scope of this straightforward and selective functionalization method.

## Introduction

Chemical functionalization of fullerenes is a fascinating and extensively studied approach, playing a pivotal role in fullerene-based materials science to introduce various characteristic functionalities [1–7]. Significant progress in synthetic procedures has contributed to diversifying their properties, enabling widespread and interdisciplinary applications in various research fields, such as biomedicine, photovoltaic devices, and materials chemistry.

Meanwhile, lithium ion-endohedral fullerenes (Li^+^@C_60_) [8], the first member of the emerging ion-endohedral fullerene family, have attracted significant attention owing to the distinctive ionic properties originating from the ion pair structure consisting of a cationic endohedral fullerene core and an external counter anion. Despite being a relatively recent addition, Li^+^@C_60_ has been the focus of intensive studies in chemistry, physics, and related interdisciplinary fields over the past 13 years [9]. A noteworthy discovery during these investigations is the significant enhancement of reactivity arising from the encapsulated Li^+^. Both experimental and theoretical approaches have diligently explored the details of reaction kinetics, quantitatively elucidating the impact of encapsulated Li^+^ on the reactivity of the outer fullerene cage as a specialized “encapsulated” Lewis acid catalyst [10–11]. While previous studies have revealed valuable insights, such as accelerated 1,3-dipolar and Diels–Alder reactions [12–13], it is noteworthy that the anticipated diverse properties resulting from the derivatization of Li^+^@C_60_ have not yet been fully realized. To further leverage the unique properties of the novel ion-endohedral fullerene, achieving diverse property tuning through chemical modification has been in high demand for its further applications, which is similar to what has been developed during the recent empty fullerene sciences.

**Scheme 1:**
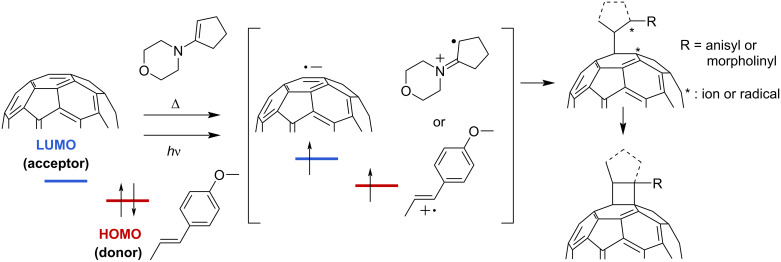
Reaction mechanisms of thermal and photoinduced [2 + 2] cycloaddition on C_60_ [19,22–23].

As a continuation of our studies on the synthesis of Li^+^@C_60_ derivatives, we herein focus on the modification of Li^+^@C_60_ through thermal [2 + 2] cycloaddition. The [2 + 2] cycloaddition reactions of empty C_60_ have been known to proceed with unsaturated substrates having HOMO levels suitable for the thermal or photoinduced single-electron-transfer (SET) process (Scheme 1) [14–23]. Although the thermal [2 + 2] reactions are generally simple and scalable, the reactions are scarcely applied for the derivatization of fullerenes due to the limitation in the variety of possible substrates. Considering the electronic effect of the encapsulated Li^+^ on the outer C_60_ cage, Li^+^@C_60_ can react with a wider range of unsaturated substrates having a relatively lower HOMO level.

With the previously uncovered reactivity of Li^+^@C_60_ in hand, we synthesized Li^+^@C_60_ derivatives in this study through the thermal [2 + 2] cycloaddition of styrene derivatives, which do not react with empty C_60_ through the same reaction pathway. Although a major issue in the derivatization of Li^+^@C_60_ is the formation of multifunctionalized byproducts, it was significantly prevented in the reaction, leading to a much better yield of the target monofunctionalized Li^+^@C_60_ derivatives [24]. Additionally, we investigated the range of the HOMO level of the reactants suitable for the thermal [2 + 2] cycloaddition of Li^+^@C_60_ using both experimental and theoretical approaches. This study clearly demonstrated the significantly improved reactivity of Li^+^@C_60_ in the thermal [2 + 2] cycloaddition reaction, highlighting the expanded scope of this straightforward and selective reaction for Li^+^@C_60_.

## Results and Discussion

**Figure 1:**
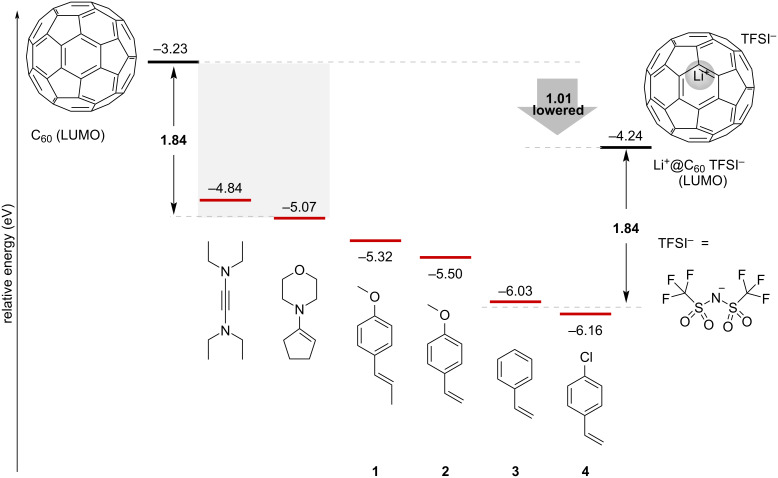
HOMO levels of unsaturated substrates and LUMO levels of fullerenes computed at the B3LYP/6-31G(d) level of theory.

We began by performing density functional theory (DFT) calculations to screen the substrates with suitable HOMO levels for the thermal [2 + 2] cycloaddition with Li^+^@C_60_. The structures of several kinds of possible reactants were optimized at the B3LYP/6-31G(d) level of theory. The calculated HOMO levels are summarized in Figure 1 along with the LUMO levels of Li^+^@C_60_ and empty C_60_ computed at the same level of theory. Among the computed substrates having a carbon–carbon unsaturated bond, thermal [2 + 2] cycloaddition of *N*,*N*,*N'*,*N'*-tetraethylethynediamine and 1-morpholino-1-cyclopentene with empty C_60_ has been reported [17,23], while electron-rich styrene derivatives **1** and **2** can react with empty C_60_ only through a photoinduced SET pathway [19,22]. From these results, the energy gap between the HOMO of the alkene substrate and the LUMO of the fullerene acceptor, where the thermal SET reaction is presumed to occur, is estimated to be approximately less than 1.84 eV. Taking these results into consideration, Li^+^@C_60_ with a LUMO level of −4.24 eV is expected to undergo thermal [2 + 2] cycloaddition with reactants having a HOMO level of −6.08 eV or higher, such as styrene derivatives **1**, **2**, and **3**.

**Scheme 2:**
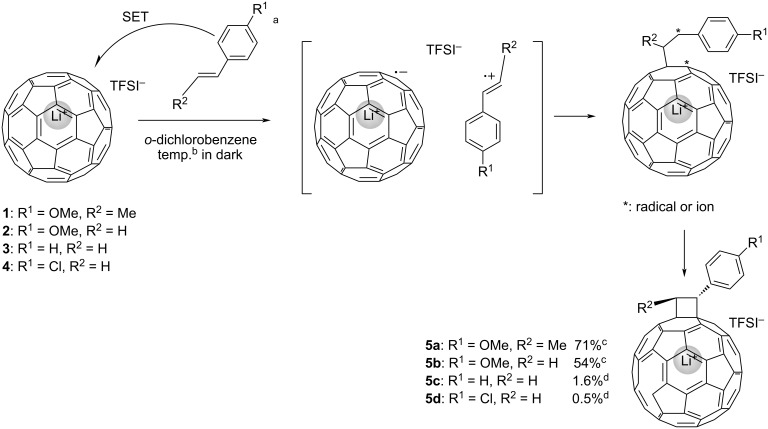
Thermal [2 + 2] reaction of Li^+^@C_60_ TFSI^−^ with substrates **1**–**4**. ^a^100 equiv for the reaction screening, 20 equiv for the synthesis of **5a**, and 40 equiv for the synthesis of **5b**. ^b^Room temperature for the reaction screening, 50 °C for the synthesis. ^c^Isolated yield. ^d^HPLC yield.

**Figure 2:**
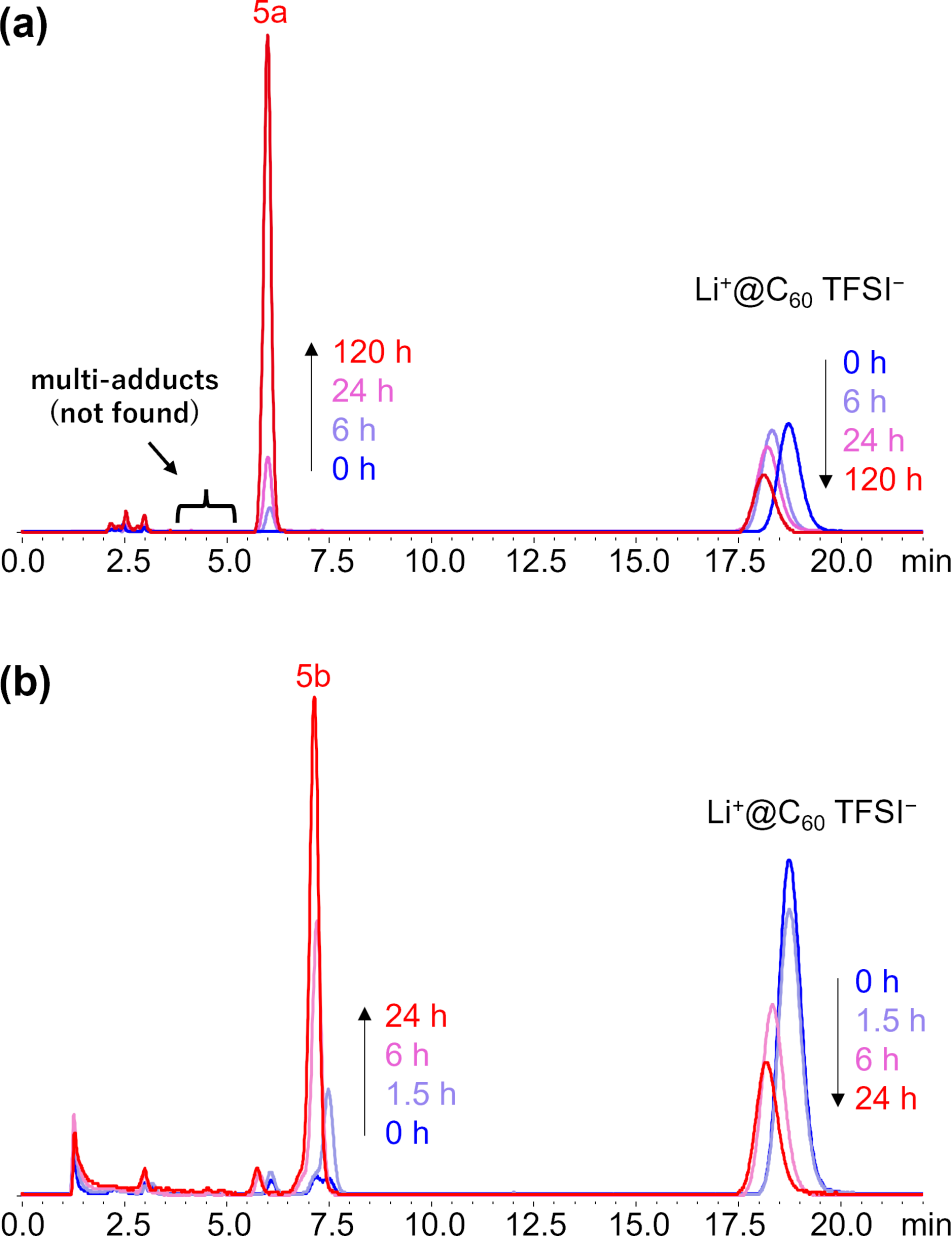
HPLC profiles of themal [2 + 2]reaction of Li^+^@C_60_ with substrate **1** (a) and **2** (b) in *o*-dichlorobenzene at room temperature.

**Figure 3:**
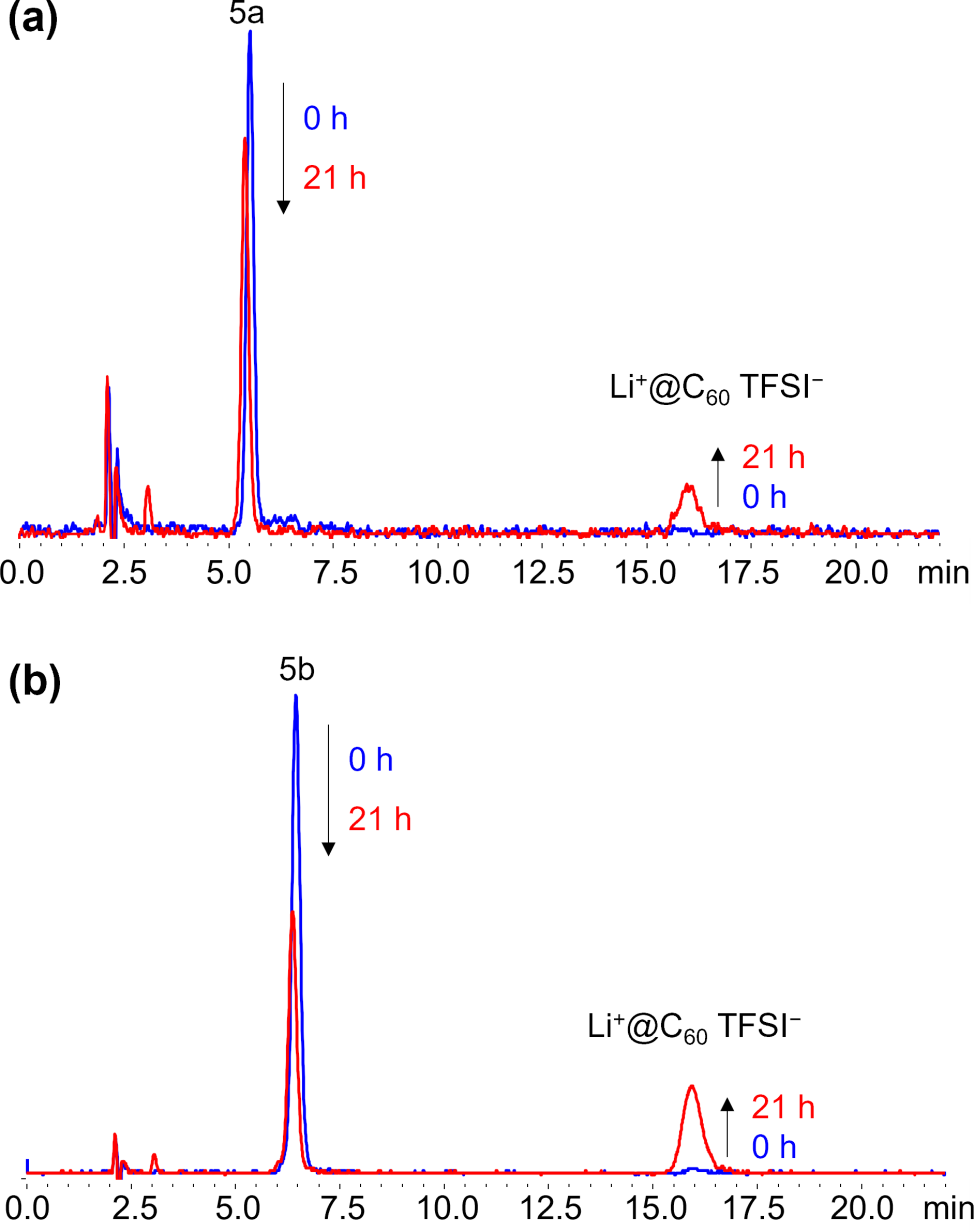
HPLC profiles of **5a** (a) and **5b** (b) before and after photoirradiation at room temperature.

Based on the results of theoretical calculations, styrene derivatives **1**, **2**, and **3** were selected as the reactants for the thermal [2 + 2] cycloaddition with Li^+^@C_60_. For comparison, we also investigated the reaction of reactant **4**, which has a larger energy gap between its HOMO and LUMO of Li^+^@C_60_ (1.92 eV). All reactions were conducted in the dark to avoid photoinduced SET reactions (Scheme 2). First, the reactivity was assessed by monitoring the reaction progress using a previously developed electrolyte-added HPLC technique [25]. As expected, both substrates **1** and **2** reacted with Li^+^@C_60_ at room temperature and exhibited HPLC signals assignable to the desirable monoadducts **5a** and **5b** (Figure 2). It is noteworthy that the reaction of **2** proceeded faster than that of **1**, although **2** has a lower HOMO level than **1**. This is likely due to the steric effect caused by the methyl group directly connected to the alkenyl C=C bond in reactant **1**. After optimizing the reaction conditions, compounds **5a** and **5b** were isolated in 71% and 53% yields, respectively. Importantly, the generation of multiadducts in the thermal [2 + 2] cycloaddition was significantly prevented, even under conditions with an excess amount of reactant, resulting in much better yields of the target products compared to other reported reactions of Li^+^@C_60_. It should also be mentioned that while these products were stable at ambient temperature in the dark, photoirradiation triggered the elimination of the addends, reforming the starting Li^+^@C_60_ (Figure 3). No other insoluble or undetectable products by HPLC were identified during the study. On the other hand, the reactions of **3** and **4** with Li^+^@C_60_ did not proceed significantly even under higher temperature reaction conditions (**5c**: 1.6% and **5d**: 0.5% in HPLC yields, Figures S1 and S2 in Supporting Information File 1). This result indicates that the HOMO levels of compounds **3** and **4** are around the threshold HOMO level for the thermal reaction with Li^+^@C_60_. The slightly higher reactivity of **3** than **4** can be simply explained by the higher HOMO level of **3** compared to that of **4**.

**Figure 4:**
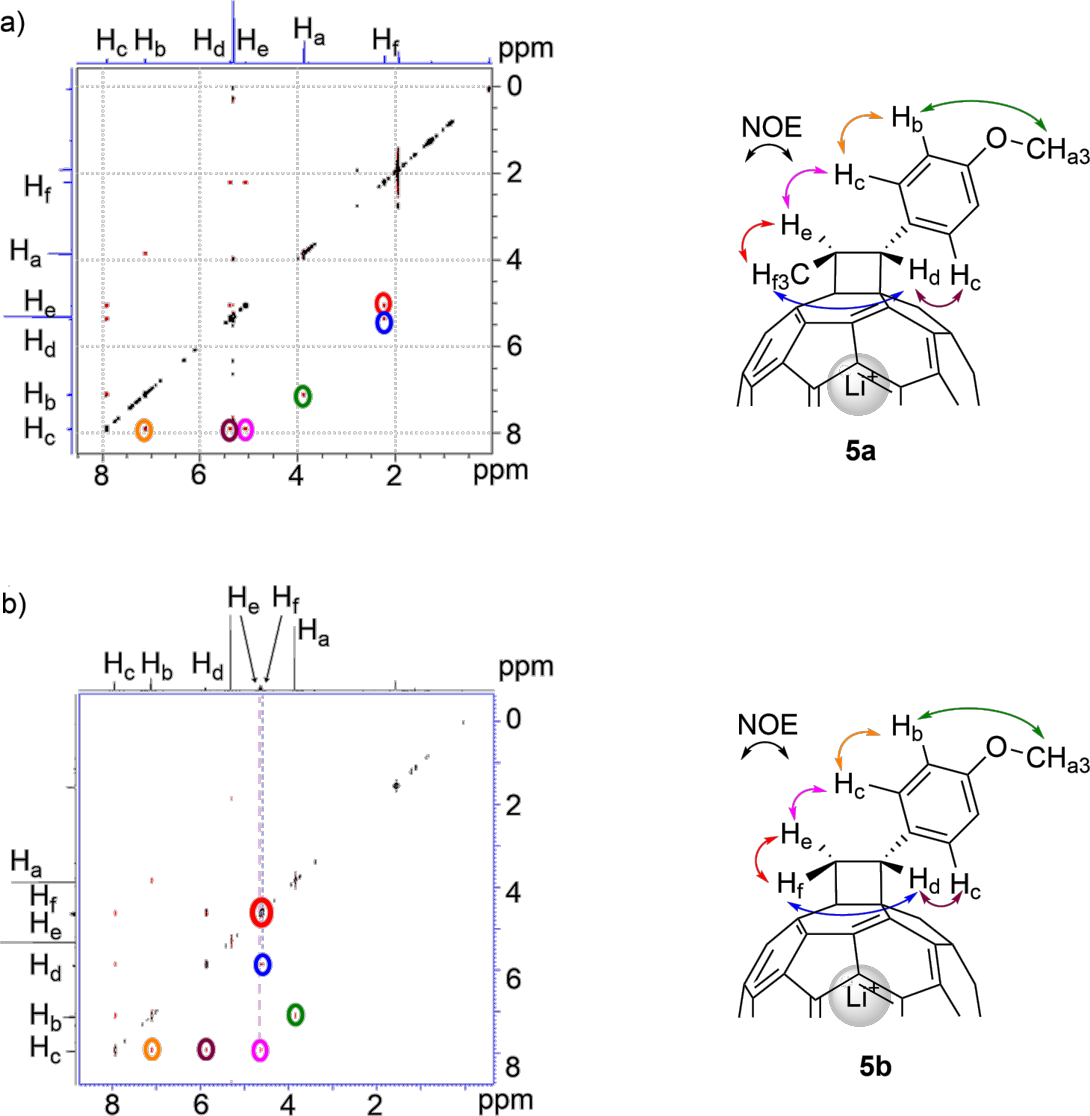
^1^H-^1^H 2D-NOESY NMR spectrum (600 MHz, CD_2_Cl_2_) of **5a** (a) and NOE correlations between two protons. The spectrum (700 MHz, CD_2_Cl_2_) of **5b** is shown in (b).

The products were characterized by spectroscopic and spectrometric analyses (Figures S3–S11 in Supporting Information File 1). ^1^H, and ^13^C NMR spectra clearly indicated the formation of [2 + 2] monoadducts. ^7^Li NMR spectra showed a sharp singlet signal at −12.4 (**5a**) and −13.5 ppm (**5b**), which clearly indicated that the Li^+^ was encapsulated in the highly shielded inner space of the fullerene cage. The observed chemical shift was almost identical to that of reported Li^+^@C_60_ derivatives [10,12,24]. Although the product **5a** may have stereoisomers, only the *E*-isomer was observed, as confirmed by ^1^H-^1^H 2D-NOESY NMR spectrum (Figure 4). This is not surprising because similar stereoselectivity has been reported in the photoinduced [2 + 2] cycloaddition reaction of empty C_60_, where the *E*-isomer is most thermodynamically stable [19,22]. The positive mode high-resolution matrix-assisted laser desorption ionization mass spectra showed the formation of the monoadducts at *m*/*z* 875.10431 (**5a**) and 861.08866 (**5b**), which were assigned to each molecular ion ([M]^+^ calcd for C_70_H_12_OLi (**5a**): 875.10427 and C_69_H_10_OLi (**5b**): 861.08862, respectively). The UV–vis absorption spectra showed broad absorption in the visible region with an absorption maximum at 711 nm, which was known to show a characteristic pattern of functionalized fullerene having an addend on a [6,6] bond [26].

**Figure 5:**
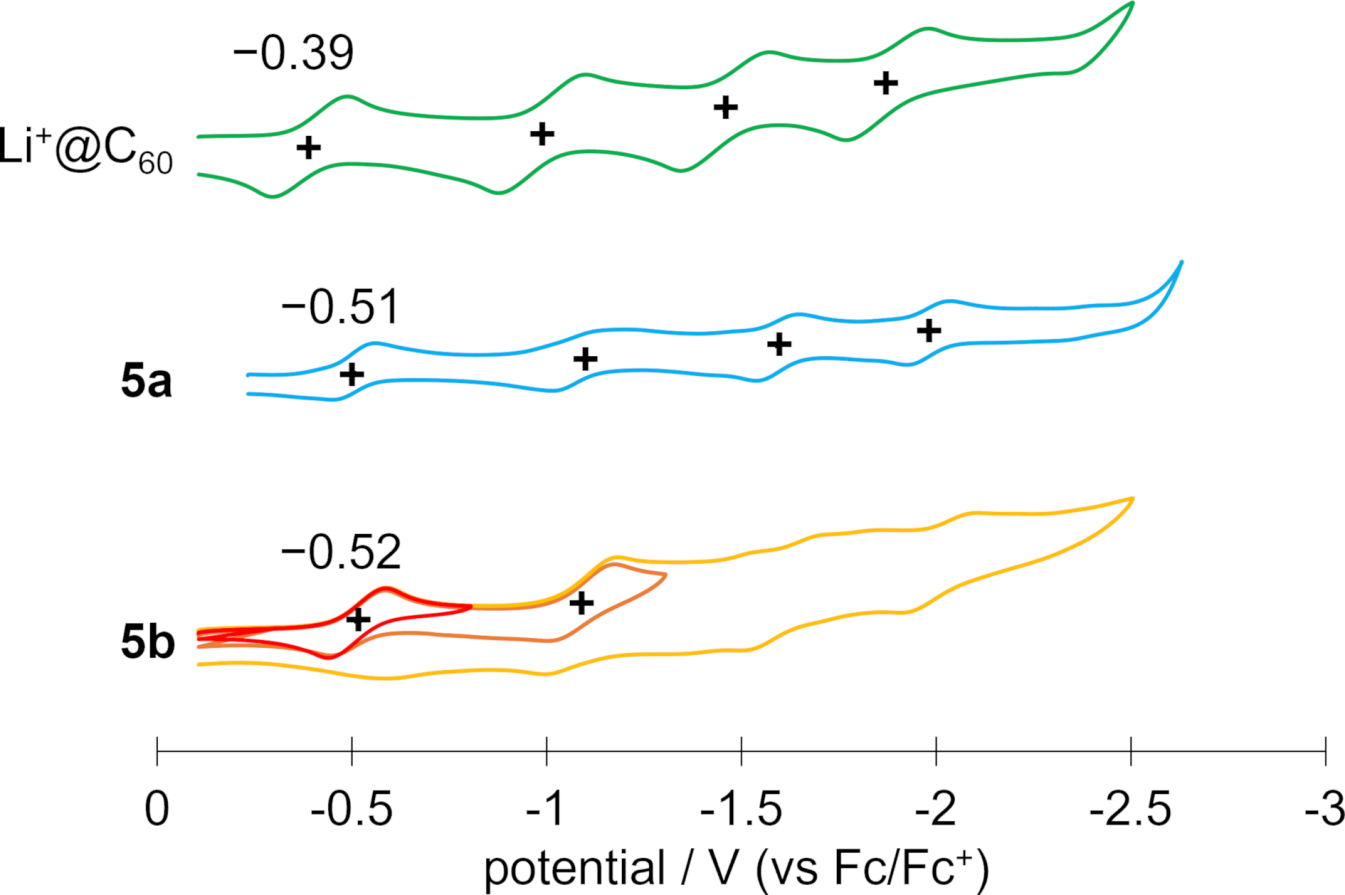
Cyclic voltammograms of **5a**, **5b**, and Li^+^@C_60_ TFSI^−^ with the potentials relative to the ferrocene/ferrocenium (Fc/Fc^+^) reference couple. Working electrode: Pt, counter electrode: Pt, reference electrode: Ag/Ag^+^ in acetonitrile, solvent: *o*-dichlorobenzene, supporting electrolyte: 50 mM TBAPF_6_.

As mentioned above, a distinctive feature of this reaction is the significantly lower yield of multiadducts compared to previously reported functionalizations of Li^+^@C_60_. The reason can be explained by the difference in electron-accepting ability between the monoadduct and pristine Li^+^@C_60_ investigated by cyclic voltammetry (Figure 5). Both products exhibited reversible first and second redox waves, with subsequent reduction resulting in an irreversible electrochemical response. The first reduction potentials of **5a** and **5b** were measured at −0.51 V and −0.52 V (vs Fc/Fc^+^), respectively, which were more negative than that of pristine Li^+^@C_60_ (*E*_1/2_^red1^ = −0.39 V). While the detailed reasons for the irreversible redox properties after the second reduction process have not been thoroughly investigated, the observed phenomena could potentially be attributed to ring opening or simple decomposition under the conditions. From these results, the LUMO levels of the compounds were estimated according to the following equation [27]: *E*_LUMO_ (eV) = −[4.80 + *E*_1/2_^red1^ (V vs Fc/Fc^+^)], and the results were summarized in Table 1. The monoadducts with a higher LUMO level are expected to have lower reactivity in the thermal [2 + 2] cycloaddition than pristine Li^+^@C_60_. Moreover, it is plausible that unreacted Li^+^@C_60_ serves as an oxidant for the reduced monoadducts potentially generated by SET from reactants to monoadducts. These factors contribute to the suppression of multiadduct formation, resulting in the selective generation of the target monoadducts. Specifically, Li^+^@C_60_, influenced by the electronic effects of the encapsulated Li^+^ Lewis acid, commonly exhibits significantly higher reactivity compared to empty C_60_. The much-enhanced reactivity often leads to the formation of multiadducts more notably than in the case of empty fullerenes, and hence, achieving the selective monofunctionalization of Li^+^@C_60_ has been a major challenge. The approach we developed in this study proves highly advantageous for the selective formation of monofunctionalized Li^+^@C_60_ derivatives, holding great promise for the design, properties tuning, and synthesis of Li^+^@C_60_-based materials for future applications.

**Table 1:** First reduction potential and estimated LUMO level of **5a** and **5b**. The values of Li^+^@C_60_ are also listed as a reference.

	*E*_1/2_^red1^ (V, vs Fc/Fc^+^)	LUMO level (eV)

**5a**	−0.51	−4.29
**5b**	−0.52	−4.28
Li^+^@C_60_ TFSI^−^	−0.39	−4.41

## Conclusion

In summary, we successfully synthesized Li^+^@C_60_ derivatives through the thermal [2 + 2] cycloaddition of styrene derivatives. Due to the lower-lying LUMO of Li^+^@C_60_, the styrene reactant, which did not react with empty C_60_ through the same pathway, underwent a reaction with Li^+^@C_60_, yielding the target monofunctionalized products. The results underscore the significantly enhanced reactivity of Li^+^@C_60_ in the thermal [2 + 2] cycloaddition reaction due to the electronic effect of the encapsulated Li^+^ Lewis acid. Moreover, the formation of undesirable bis- and multiadducts was notably suppressed, resulting in much better yields of the target monoadducts. Electrochemical measurements revealed that the functionalization raised the LUMO level of Li^+^@C_60_, leading to lower reactivity for the second addition. With this facile, selective, and high-yield approach for the derivatization of ion-endohedral fullerene, the design and synthesis of novel Li^+^@C_60_ derivatives for further application in various research fields are currently underway.

## Experimental

### General procedure

Unless otherwise noted, all chemicals, including anhydrous solvents, were obtained from commercial suppliers (FUJIFILM Wako Pure Chemical Corp., TCI, Sigma-Aldrich) and used as received without further purification. Li^+^@C_60_TFSI^−^ was purchased as PF_6_^−^ salt from Idea International Corp., and then its counter anion was exchanged to TFSI^−^ according to reported procedures [9].

NMR spectra were recorded on a JEOL JNM-ECZ400S (^1^H: 400 MHz, ^7^Li: 155 MHz, ^13^C: 100 MHz), a Bruker ADVANCE III (^1^H: 600 MHz) and a Bruker ADVANCE III 700 (^1^H: 700 MHz, ^7^Li: 272 MHz, ^13^C: 176 MHz) spectrometer. Chemical shifts (δ) were reported in parts per million (ppm) relative to residual proton of solvent for ^1^H (5.32 ppm, CDHCl_2_), LiCl in D_2_O for ^7^Li (0 ppm, external standard), and carbon of the solvent for ^13^C (53.84 ppm, CD_2_Cl_2_). High-resolution matrix-assisted laser desorption ionization (HR-MALDI) mass spectra were obtained on a Bruker solariX 12T mass spectrometer with dithranol as a matrix. UV–vis absorption spectra were measured on a JASCO V-670 and a Shimadzu UV-1800 spectrophotometer. Cyclic voltammograms were recorded on a BAS ALS 600A and a BAS ALS 620D apparatus with a three-electrode system.

### Reactivitiy comparison of Li^+^@C_60_ TFSI^−^ and styrenes

In an Ar-filled glove box, Li^+^@C_60_ TFSI^−^ and styrenes **1**–**4** were dissolved in anhydrous chlorobenzene. 100 µL of Li^+^@C_60_ TFSI^−^ solution (2.0 mM) and 100 μL of each styrene solution (200 mM) were mixed, respectively. The solutions were stirred in glove box for the indicated reaction time. At the time, 20 μL of solution was divided, taken out from glove box, frozen by liq. N_2_, and stored in a freezer until HPLC measurement.

The solutions were subjected to analytical HPLC. HPLC profiles are shown in Supporting Information File 1. HPLC conditions: column: Buckyprep ø 4.6 × (10 + 250) mm; mobile phase: chlorobenzene/acetonitrile 95:5 containing 30 mM LiTFSI; flow rate: 1.5 mL/min; temperature: 50 °C; detector: UV 337 nm; injection sample volume: 5 µL.

### Synthesis of Li^+^@C_60_{(4-MeOC_6_H_4_)CH=CHMe} TFSI^−^ (**5a**)

To 2.5 mL of a chlorobenzene/acetonitrile 1:1 (v/v) solution containing Li^+^@C_60_ TFSI^−^ (8.5 mg, 8.4 µmol) was added *trans*-anethole (25 µL, 24.7 mg, 0.17 mmol). The solution was stirred at 50 °C for 15 hours. The resulted solution was subjected to preparative HPLC. Condition: solvent: chlorobenzene/acetonitrile 4:11 (v/v) containing 2 mM LiTFSI; column: Inertsil CX (GL Sciences), ø 4.6 × 250 mm. The fraction containing Li^+^@C_60_{(4-MeOC_6_H_4_)CH=CHMe} was collected and evaporated under reduced pressure. The resulting solid was washed with diethyl ether and dissolved in dichloromethane. The desired monoadduct Li^+^@C_60_{(4-MeOC_6_H_4_)CH=CHMe} TFSI^−^ (**5a**, 6.9 mg, 6.0 µmol, 71%) was afforded from the solution by vapor-diffusion recrystallization with diethyl ether.

^1^H NMR (400 MHz, CD_2_Cl_2_) δ 2.23 (d, *J* = 6.9 Hz, 3H), 3.88 (s, 3H), 5.07 (qd, *J* = 6.9 8.7 Hz, 1H), 5.38 (d, *J* = 8.7 Hz, 1H), 7.12 (d, *J* = 8.7 Hz, 2H), 7.92 (d, *J* = 8.8 Hz, 2H); ^13^C NMR (100 MHz, CD_2_Cl_2_) δ 19.83, 47.39, 55.80, 58.72, 70.58, 73.03, 115.10, 119.84 (q, *J*_CF_ = 320 Hz, CF_3_), 129.96, 130.56, 136.89, 136.93, 139.24, 139.66, 140.23, 140.50, 141.45, 141.62, 141.70, 141.79, 141.82, 141.85, 142.02, 142.34, 142.50, 142.53, 142.76, 142.84, 142.85, 142.92, 142.96, 143.00, 143.19, 144.04, 144.24, 144.32, 144.80, 144.89, 144.93, 145.02, 145.20, 145.23, 145.35, 145.39, 145.50, 145.57, 145.62, 145.79, 145.82, 145.91, 145.96, 145.99, 146.05, 146.12, 146.66, 146.72, 147.48, 147.85, 153.12, 153.33, 156.27, 156.48, 160.24; ^7^Li NMR (155 MHz, CD_2_Cl_2_) δ −12.4; HRMS–MALDI–TOF, positive ion mode, dithranol (*m*/*z*): [M]^+^ calcd for C_70_H_12_OLi, 875.10427; found, 875.10431.

### Synthesis of Li^+^@C_60_{(4-MeOC_6_H_4_)CH=CH_2_} TFSI^−^ (**5b**)

To 2.5 mL of a chlorobenzene/acetonitrile 1:1 (v/v) solution containing Li^+^@C_60_ TFSI^−^ (9.4 mg, 9.3 µmol) was added 4-methoxystyrene (50.1 µL, 50.1 mg, 0.37 mmol). The solution was stirred at 50 °C for 45 min. The resulted solution was subjected to preparative HPLC. Conditions: solvent: chlorobenzene/acetonitrile 95:5 (v/v) containing 30 mM LiTFSI; column: Buckyprep (Nacalai tesque), ø 10 × (20 + 250) mm. The fraction containing Li^+^@C_60_{(4-MeOC_6_H_4_)CH=CH_2_} was concentrated under reduced pressure. The desired monoadduct Li^+^@C_60_{(4-MeOC_6_H_4_)CH=CH_2_} TFSI^−^ (**5b**, 5.6 mg, 4.9 µmol, 53%) was afforded by precipitation with diethyl ether and filtration.

^1^H NMR (700 MHz, CD_2_Cl_2_) δ 3.87 (s, 3H), 4.59 (dd, *J* = 10.8 Hz, 13.8 Hz, 1H), 4.69 (dd, *J* = 8.6 Hz, 13.8 Hz, 1H), 5.89 (dd, *J* = 8.6, 10.7 Hz, 1H), 7.12 (d, *J* = 8.8 Hz, 2H), 7.95 (d, *J* = 8.7 Hz, 2H); ^13^C NMR (176 MHz, CD_2_Cl_2_) δ 37.94, 49.75, 55.65, 64.70, 74.90, 114.85, 120.07 (q, *J*_CF_ = 321 Hz, CF_3_), 129.69, 131.42, 136.98, 137.32, 137.89 ,138.88, 139.99, 140.14, 140.24, 140.27, 141.24, 141.34, 141.55, 141.57, 141.60, 141.66, 141.68, 141.71, 142.18, 142.23, 142.35, 142.37, 142.61, 142.65, 142.66, 142.73, 142.76, 142.80, 143.01, 143.90, 144.02, 144.06, 144.09, 144.73, 144.83, 144.90, 144.94, 145.02, 145.14, 145.22, 145.29, 145.43, 145.57, 145.61, 145.63, 145.67, 145.72, 145.77, 145.82, 145.90, 146.51, 146.69, 146.85, 153.12, 155.45, 155.75, 155.85, 159.84; ^7^Li NMR (272 MHz, CD_2_Cl_2_) δ −13.3; HRMS–MALDI–TOF, positive ion mode, dithranol (*m*/*z*): [M]^+^ calcd for C_69_H_10_OLi, 861.08862; found, 861.08866.

## Supporting Information

File 1 HPLC profiles, NMR, HRMS, UV–vis absorption spectra, and computational details.

## Data Availability

The data that supports the findings of this study is available from the corresponding author upon reasonable request.
