# Supplementary material for: Enhanced reactivity of Li+@C60 toward thermal [2 + 2] cycloaddition by encapsulated Li+ Lewis acid
Source: Beilstein J Org Chem. 2024 Mar 25;20:653–60. doi: 10.3762/bjoc.20.58 (PMC10999987; doi:10.3762/bjoc.20.58)
Supplement: File 1 — HPLC profiles, NMR, HRMS, UV–vis absorption spectra, and computational details. [file Beilstein_J_Org_Chem-20-653-s001.pdf]

BEILSTEIN JOURNAL OF ORGANIC CHEMISTRY

Supporting Information

for

Enhanced reactivity of $Li^+@C_{60}$ toward thermal [2 + 2] cycloaddition by encapsulated Li^+ Lewis acid

Hiroshi Ueno, Yu Yamazaki, Hiroshi Okada, Fuminori Misaizu, Ken Kokubo and Hidehiro Sakurai

Beilstein J. Org. Chem. **2024,** *20,* 653–660. doi:10.3762/bjoc.20.58

HPLC profiles, NMR, HRMS, UV–vis absorption spectra, and computational details

License and Terms: This is a supporting information file under the terms of the Creative Commons Attribution License (https://creativecommons.org/licenses/by/4.0). Please note that the reuse, redistribution and reproduction in particular requires that the author(s) and source are credited and that individual graphics may be subject to special legal provisions.
The license is subject to the *Beilstein Journal of Organic Chemistry* terms and conditions: (https://www.beilstein-journals.org/bjoc/terms)

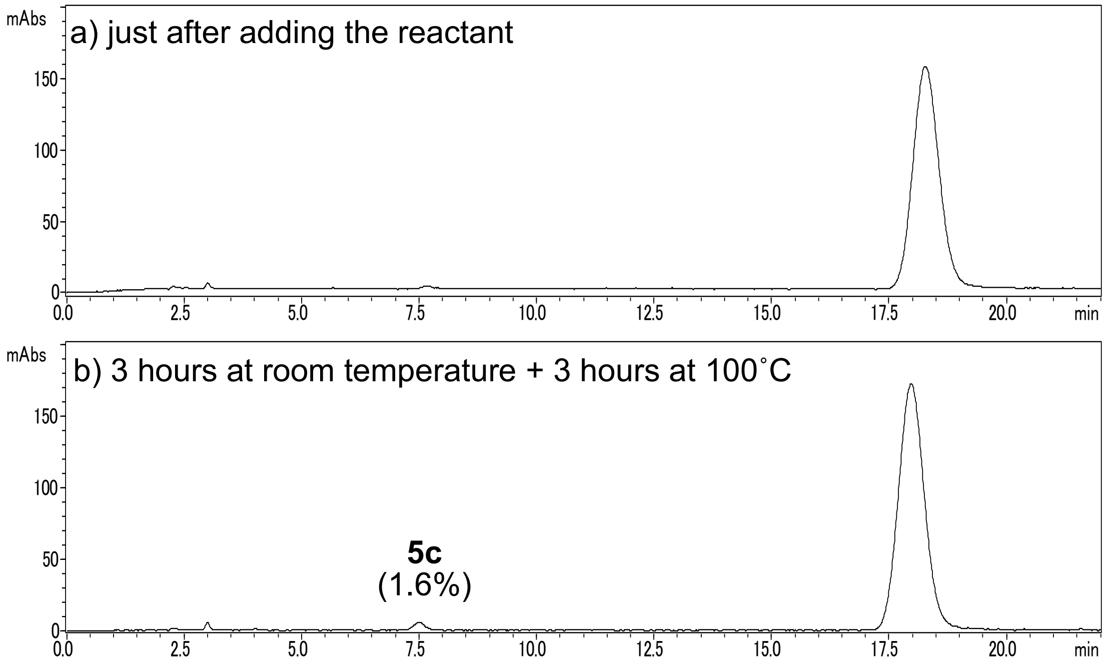

Figure S1: HPLC profiles for the reaction of $Li^+@C_{60}$ $TFSI^-$ (1 mM) and **3** (100 mM) in ODCB. a) just after adding the reactant, b) 3 hours at room temperature + 3 hours at 100 °C.

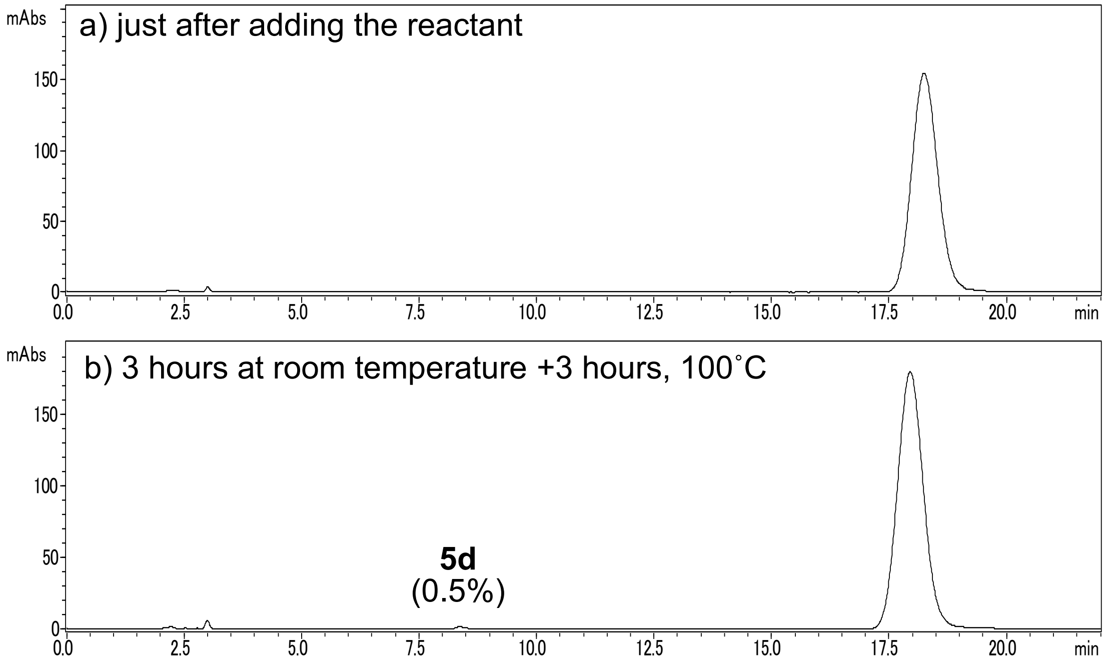

Figure S2: HPLC profiles for the reaction of $Li^+@C_{60}$ $TFSI^-$ (1 mM) and **4** (100 mM) in ODCB. a) just after adding the reactant, b) 3 hours at room temperature + 3 hours at 100 °C.

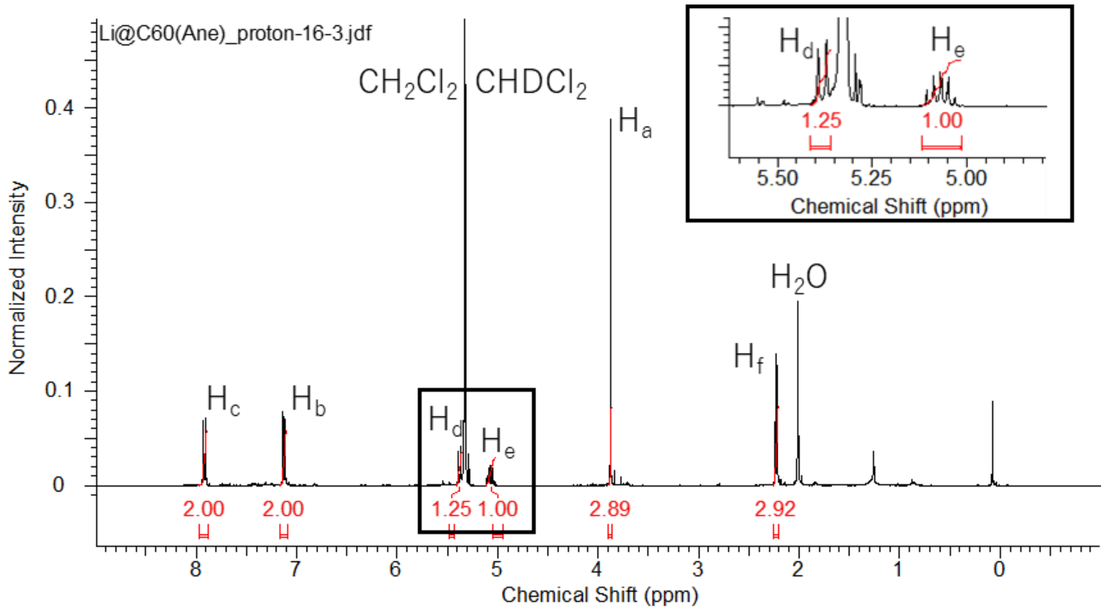

Figure S3: ¹H NMR spectrum (400 MHz, CD_2Cl_2) of **5a**.

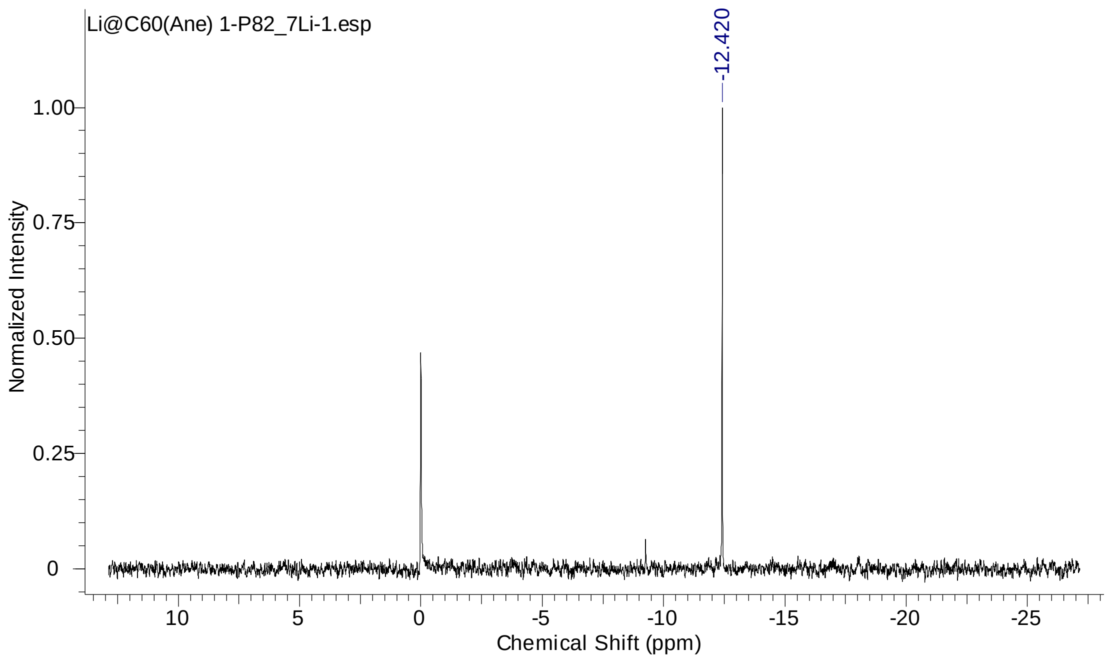

Figure S4: ⁷Li NMR spectrum (155 MHz, CD_2Cl_2) of **5a**. LiCl in D_2O was used as an external standard (0 ppm)

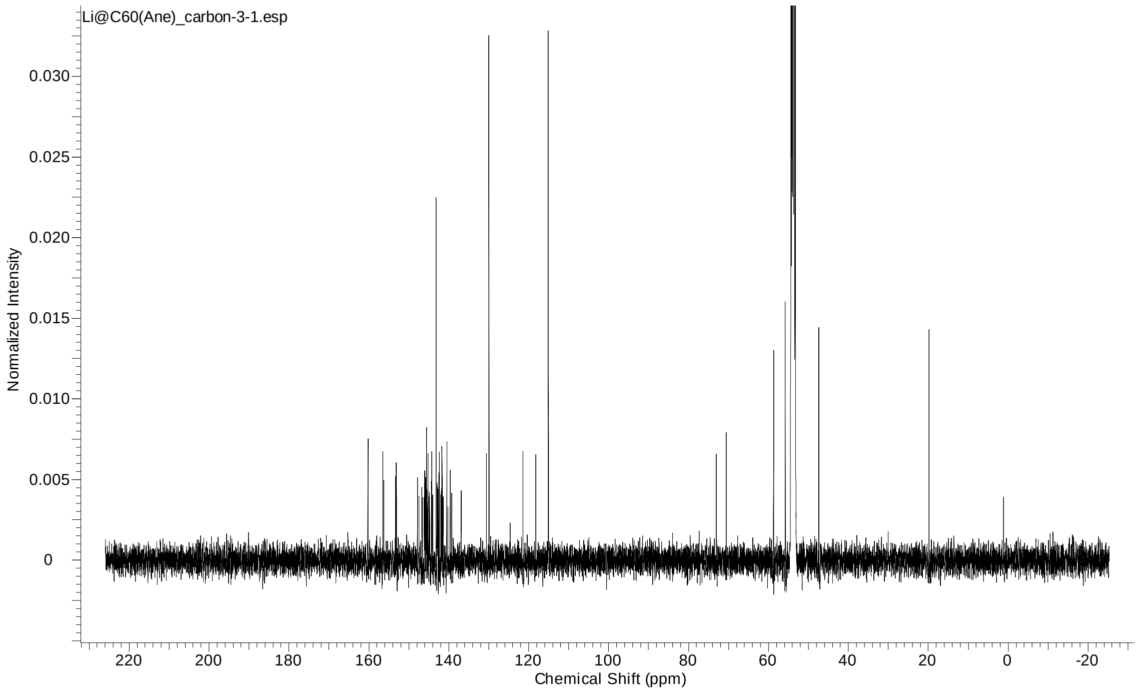

Figure S5: ^{13}C NMR spectrum (100 MHz, CD_2Cl_2) of **5a**.

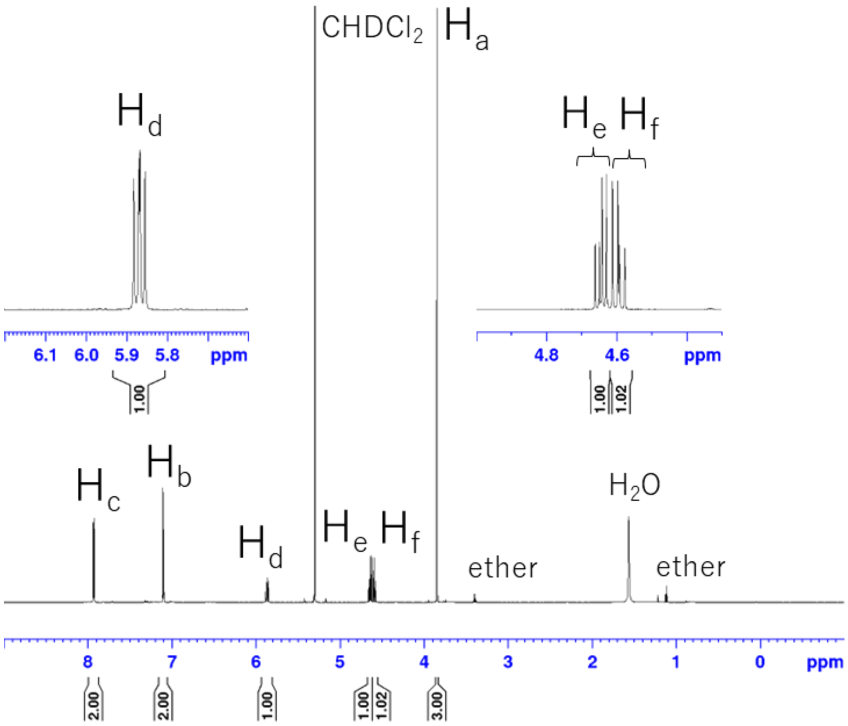

Figure S6: ^{1}H NMR spectrum (700 MHz, CD_2Cl_2) of **5b**.

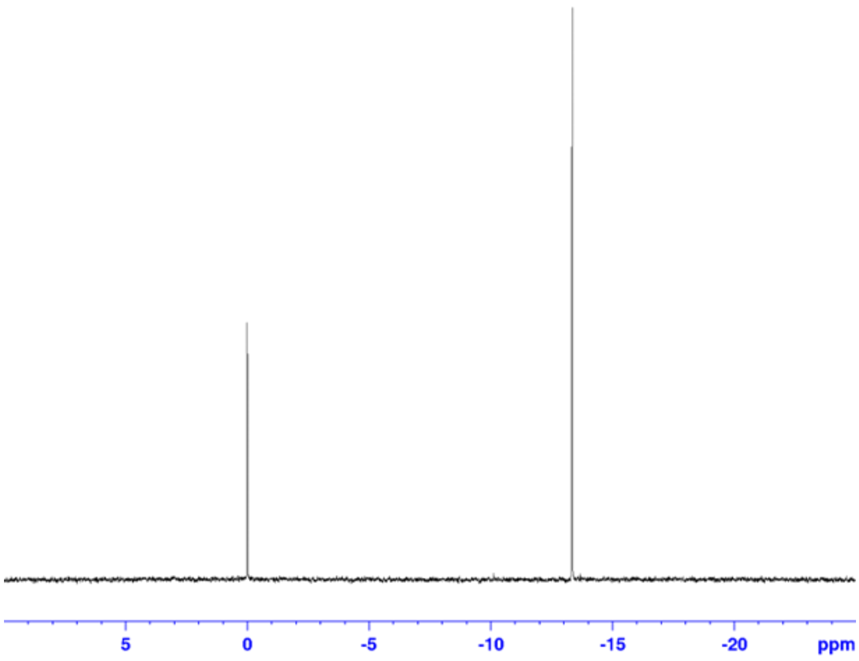

Figure S7: ^{7}Li NMR spectrum (272 MHz, CD_2Cl_2) of **5b**. LiCl in D_2O was used as an external standard (0 ppm).

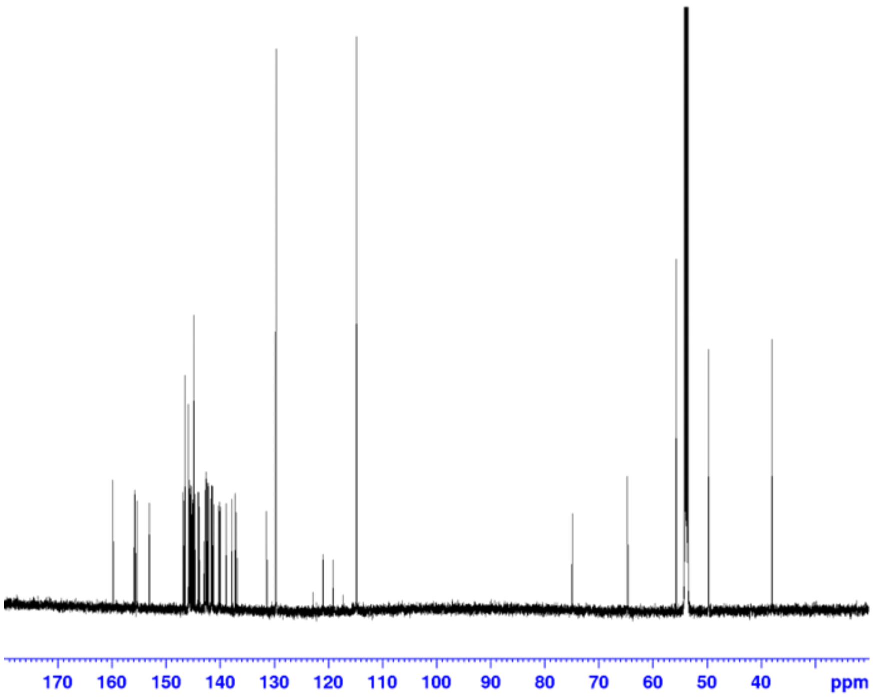

Figure S8: ^{13}C NMR spectrum (176 MHz, CD_2Cl_2) of **5b**.

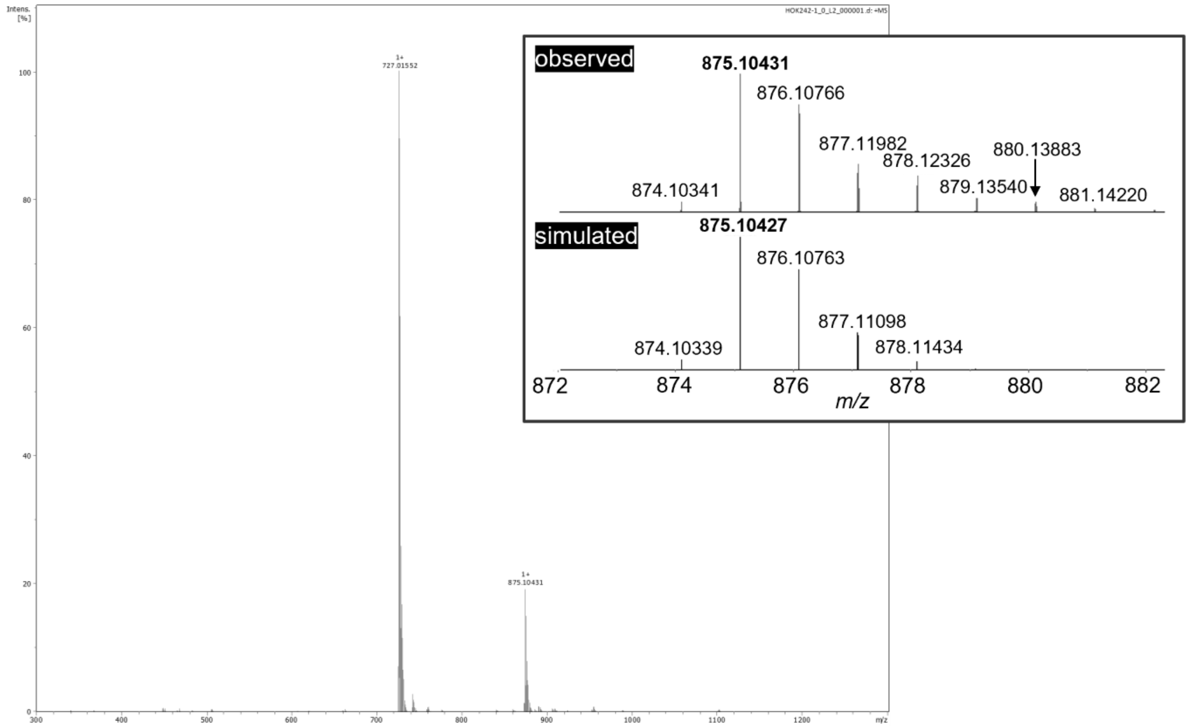

Figure S9: HRMS-MALDI (positive, dithranol) spectrum of **5a**.

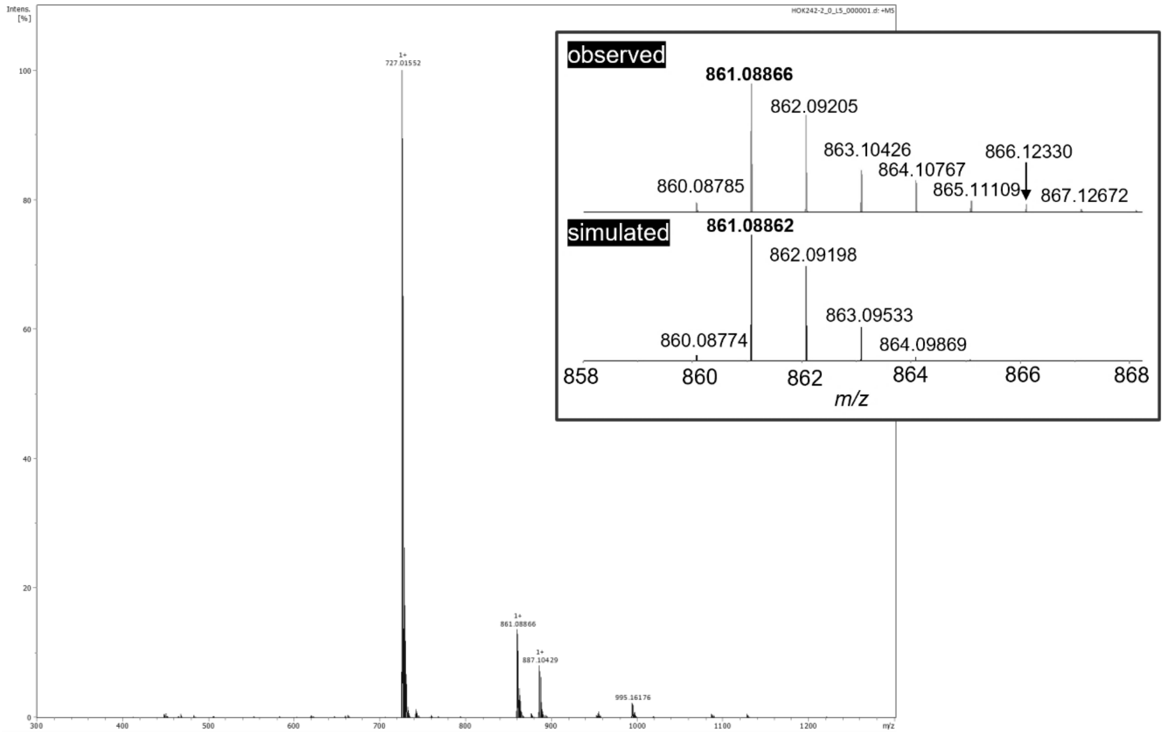

Figure S10: HRMS-MALDI (positive, dithranol) spectrum of **5b**.

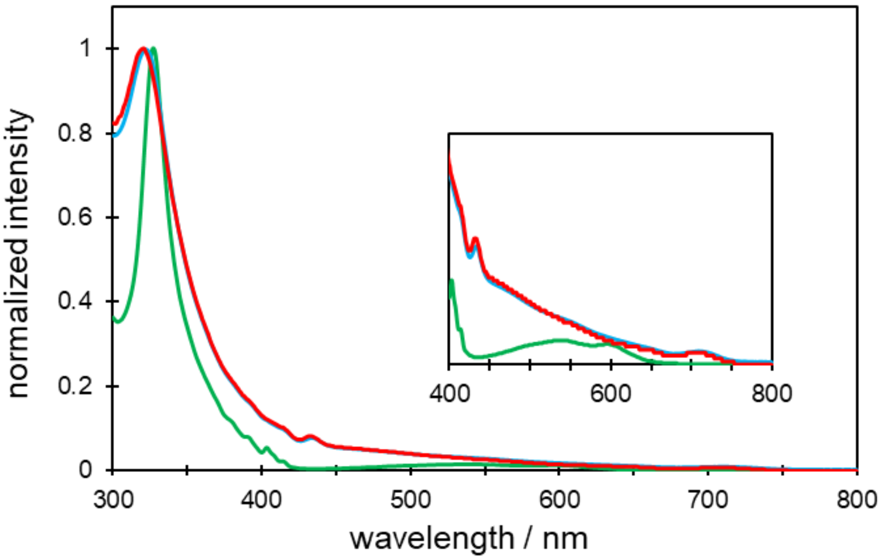

Figure S11: UV–vis absorption spectra of **5a** (blue), **5b** (red), and $Li^{+}@C_{60}$ $TFSI^{-}$ (green) measured in dichloromethane.

Computational details

Full geometry optimizations followed by frequency calculations have been carried out by using Gaussian 16 program at the B3LYP/6-31g(d) level. All frequency calculations showed no imaginary frequency. Full citation is as follows:

Gaussian 16, Revision C.01, M. J. Frisch, G. W. Trucks, H. B. Schlegel, G. E. Scuseria, M. A. Robb, J. R. Cheeseman, G. Scalmani, V. Barone, G. A. Petersson, H. Nakatsuji, X. Li, M. Caricato, A. V. Marenich, J. Bloino, B. G. Janesko, R. Gomperts, B. Mennucci, H. P. Hratchian, J. V. Ortiz, A. F. Izmaylov, J. L. Sonnenberg, D. Williams-Young, F. Ding, F. Lipparini, F. Egidi, J. Goings, B. Peng, A. Petrone, T. Henderson, D. Ranasinghe, V. G. Zakrzewski, J. Gao, N. Rega, G. Zheng, W. Liang, M. Hada, M. Ehara, K. Toyota, R. Fukuda, J. Hasegawa, M. Ishida, T. Nakajima, Y. Honda, O. Kitao, H. Nakai, T. Vreven, K. Throssell, J. A. Montgomery, Jr., J. E. Peralta, F. Ogliaro, M. J. Bearpark, J. J. Heyd, E. N. Brothers, K. N. Kudin, V. N. Staroverov, T. A. Keith, R. Kobayashi, J. Normand, K. Raghavachari, A. P. Rendell, J. C. Burant, S. S. Iyengar, J. Tomasi, M. Cossi, J. M. Millam, M. Klene, C. Adamo, R. Cammi, J. W. Ochterski, R. L. Martin, K. Morokuma, O. Farkas, J. B. Foresman, and D. J. Fox, Gaussian, Inc., Wallingford CT, 2019.

Summary of calculated energies

	E / Hartree	LUMO level	
		Hartree	eV
$Li^{+}@C_{60}$ $TFSI^{-}$	−4120.8113	−0.15594	−4.24
C_{60}	−2286.1742	−0.11853	−3.23

	E / Hartree	HOMO level	
		Hartree	eV
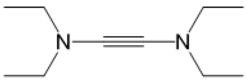	−502.52977	−0.17798	−4.84
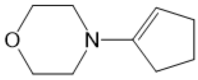	−481.93011	−0.18645	−5.07
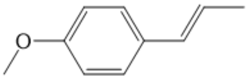	−463.49085	−0.19554	−5.32
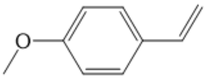	−424.1714	−0.20227	−5.50
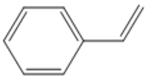	−309.64825	−0.22166	−6.03
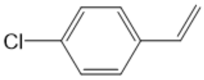	−769.24449	−0.2264	−6.16

Calculated geometrical coordinates

$Li^{+}@C_{60}$ $TFSI^{-}$

	x	y	z
C	2.42566000	-1.78500400	9.75527700
C	1.64308700	-2.40059900	8.69549700
C	0.52195800	-3.09417700	9.30863000
C	0.61204100	-2.90706600	10.74809400
C	1.78922000	-2.09952700	11.02425200
C	3.00400700	-0.52984000	9.55732100
C	2.82582700	0.16196400	8.29098800
C	2.07370600	-0.42852600	7.27451400
C	1.46986800	-1.73483400	7.48135900
C	0.17066100	-1.73735100	6.82852500
C	-0.90525300	-2.40425300	7.41610100
C	-0.72598100	-3.09477500	8.68301900
C	-1.93581500	-2.91109100	9.46862200
C	-1.85100600	-2.73455700	10.85211400
C	-0.55074100	-2.73280000	11.50500800
C	-0.58511500	-1.74446300	12.57724200
C	0.55117100	-0.96789100	12.84460200
C	1.75809100	-1.14637400	12.04715500
C	2.36197000	0.16201200	11.84009400
C	2.97048200	0.46299100	10.61905200
C	2.77188900	1.76759700	10.00881700
C	2.68316500	1.58146300	8.56984800
C	1.14795800	0.37557400	6.49395400
C	-0.02778000	-0.43333900	6.21807100
C	-2.22492200	-1.79440700	7.41946100
C	-2.86213500	-2.10845000	8.68787900
C	-2.68806400	-1.74428800	11.51280300
C	-1.91073800	-1.13213700	12.58433800
C	0.40521300	0.45996300	13.12258400
C	1.52714400	1.15380800	12.49984300
C	1.79260800	2.35295400	7.82177600
C	1.01025800	1.73818000	6.76202300
C	-1.29430400	0.15271400	6.22212300
C	-2.41493300	-0.54122100	6.83520200
C	-3.66399700	-1.15636800	9.32163700
C	-3.57725500	-0.97162200	10.76048900
C	-2.05391100	0.23581000	12.86355400
C	-0.86635300	1.04778000	13.12486500
C	1.33294000	2.40596900	11.90992000
C	1.96932200	2.71937300	10.64106800
C	-1.06414100	2.35054300	12.50283100
C	0.01250200	3.01615100	11.91343400
C	1.04314300	3.52500900	9.86090400
C	0.95713700	3.34448900	8.47936800
C	-0.30884200	2.34888300	6.76513400
C	-1.43769800	1.57229600	6.50070800
C	-3.25059400	0.44958300	7.49364400
C	-3.86295500	0.14916000	8.71159300
C	-3.71624100	0.45011400	11.04015900
C	-2.96904900	1.04146300	12.06002200
C	-2.36293400	2.34576400	11.84938900
C	-0.16504700	3.71034300	10.64659100
C	-0.34152900	3.34135600	7.82682800
C	-2.64644900	1.75535200	7.28707300
C	-3.89664600	1.14127600	9.77265800
C	-3.31550400	2.39472400	9.57481800
C	-2.53366400	3.00988900	10.63509900
C	-1.41208600	3.70408500	10.02072700
C	-1.50222200	3.51731000	8.58213400
C	-2.67829400	2.70811300	8.30664200
Li	-0.62267400	0.00550100	11.14560700
N	-2.86096300	0.60453200	15.40196000
S	-1.91833700	-0.20152300	16.44646700
S	-3.67935600	1.97527300	15.71151000
O	-3.41323900	2.92713600	14.61840500
C	-5.41907300	1.38271000	15.36232200
O	-3.69061800	2.44812300	17.09519800

(continues)

	x	y	z				
				F	-3.82090900	-2.04424500	16.56948400
C	-3.09768600	-1.29110400	17.40900200	F	-2.39785000	-2.09752300	18.21525500
O	-1.13999700	-1.15025900	15.63085200	F	-5.51958100	0.94221500	14.09543100
O	-1.22976500	0.61246200	17.44758500	F	-5.75754000	0.38752400	16.18735800
F	-3.92675100	-0.54884600	18.14635900	F	-6.27598800	2.39555300	15.52814000

C_{60}

	x	y	z				
C	-0.29735800	4.99508100	2.82955000	C	-4.72430700	5.55831500	5.49977200
C	-1.03492400	4.38629000	1.73521000	C	-2.45969600	7.16331600	6.14146700
C	-2.16669600	3.67036100	2.29982000	C	-1.32784600	7.87921500	5.57689900
C	-2.12858300	3.83673000	3.74314100	C	-0.91302600	9.15075300	0.93235900
C	-0.97327600	4.65543600	4.07056200	C	-1.65046200	8.54193800	-0.16211000
C	0.27296600	6.25849900	2.66885300	C	-3.91506400	6.93693700	-0.80380000
C	0.12946600	6.96542100	1.40712800	C	-5.04691300	6.22103000	-0.23922600
C	-0.57855100	6.38081400	0.35625200	C	-6.37183100	5.55529400	2.19343000
C	-1.17254300	5.06494300	0.52362300	C	-6.33364400	5.72174300	3.63670300
C	-2.44788400	5.05601600	-0.17336700	C	-4.88803500	6.91516800	5.78180300
C	-3.53459300	4.36865300	0.36879300	C	-3.73277500	7.73397900	6.10917100
C	-3.39115800	3.66175300	1.63055400	C	-1.51431800	9.13721400	5.00238000
C	-4.62836500	3.81902400	2.37670900	C	-0.83831200	9.47688500	3.76144600
C	-4.59176200	3.97880200	3.76256100	C	-3.92687000	9.04422400	5.51102000
C	-3.31649800	3.98787100	4.45963500	C	-2.84016300	9.73158900	4.96886700
C	-3.39842100	4.96395600	5.53335600	C	-1.74641100	10.28122800	2.96094500
C	-2.28914200	5.75019000	5.84763000	C	-1.78300700	10.12146300	1.57509100
C	-1.05194500	5.59275000	5.10140000	C	-2.97633800	9.13628300	-0.19569400
C	-0.45790600	6.90858300	4.93401400	C	-4.08561600	8.35004800	-0.50997100
C	0.19109400	7.23469300	3.74243400	C	-5.91687100	7.19166100	0.40364500
C	-0.00295100	8.54494400	3.14423600	C	-6.56585600	6.86555400	1.59522700
C	-0.04112400	8.37850000	1.70094700	C	-6.50423600	7.13482500	3.93052700
C	-1.48672600	7.18507200	-0.44415000	C	-5.79620800	7.71944100	4.98139800
C	-2.64198300	6.36627400	-0.77151400	C	-5.20221900	9.03528900	4.81402400
C	-4.86044800	4.96303100	0.33527200	C	-2.98359700	10.43850200	3.70709300
C	-5.53644000	4.62336600	1.57621900	C	-3.05826100	10.11238500	0.87802800
C	-5.46174300	4.94948800	4.40529800	C	-5.32282200	8.50749600	0.23627300

(continues)

	X	y	z				
C	-6.64774800	7.84174700	2.66881100	C	-4.20805800	10.42988600	3.03782400
C	-6.07739800	9.10516200	2.50811100	C	-4.24617700	10.26352800	1.59452300
C	-5.33984600	9.71395200	3.60244100	C	-5.40150000	9.44481000	1.26709000

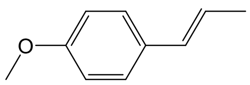

	x	y	z				
C	0.07590000	-0.04400000	0.16460000	C	2.34610000	-1.15310000	0.18740000
C	1.54430000	-0.07740000	0.20520000	H	2.01730000	0.90500000	0.25580000
C	-0.58240000	1.19350000	0.19250000	H	1.91230000	-2.15200000	0.13730000
C	-0.73200000	-1.19880000	0.09790000	C	3.84500000	-1.09650000	0.23220000
C	-2.11400000	-1.11370000	0.06170000	C	-4.81020000	1.34620000	0.07740000
C	-1.97450000	1.29910000	0.15670000	H	-4.60480000	1.90570000	0.99980000
C	-2.75060000	0.13820000	0.09070000	H	-4.55380000	1.97300000	-0.78720000
H	-0.27030000	-2.18170000	0.07400000	H	-5.87170000	1.09460000	0.03760000
H	-2.73090000	-2.00570000	0.01040000	H	4.29030000	-1.57000000	-0.65410000
H	0.00920000	2.10500000	0.24380000	H	4.20730000	-0.06390000	0.28140000
H	-2.43440000	2.28050000	0.18060000	H	4.24030000	-1.63610000	1.10420000
O	-4.11530000	0.11050000	0.05070000				

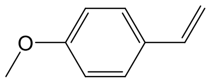

	x	y	z				
C	0.10835900	-0.05008700	0.10958700	H	-2.39896200	2.27555800	0.05547200
C	1.57734100	-0.08849900	0.10073500	O	-4.08014600	0.10472800	0.13360100
C	-0.54679200	1.18837700	0.07606500	C	2.37174400	-1.16683100	0.12934200
C	-0.69910700	-1.20595000	0.15056400	H	2.05175800	0.89285100	0.06656700
C	-2.08104500	-1.12086100	0.15744600	H	1.98788400	-2.18298300	0.16410700
C	-1.93913800	1.29423600	0.08240500	H	3.45237700	-1.06339000	0.11866700
C	-2.71600000	0.13241500	0.12338900	C	-4.77586300	1.34047700	0.10050000
H	-0.23563700	-2.18787500	0.17742400	H	-4.53869900	1.96090400	0.97498200
H	-2.69980800	-2.01240500	0.18899600	H	-4.55091400	1.90613400	-0.81346100
H	0.04656400	2.09958600	0.04403800	H	-5.83750700	1.08720400	0.11550400

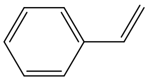

	x	y	z
C	-0.00950000	-0.04083200	0.08075400
C	1.46121900	-0.01626700	0.14392500
C	-0.70440900	1.17594300	-0.02650100
C	-0.76291300	-1.22864900	0.12242800
C	-2.15253500	-1.19590200	0.05928000
C	-2.09689900	1.21124100	-0.08993100
C	-2.82788800	0.02406800	-0.04725300
H	-0.25706000	-2.18631100	0.20486600
H	-2.71378000	-2.12615800	0.09314900
H	-0.14010600	2.10533900	-0.06024000
H	-2.60949500	2.16605800	-0.17262700
H	-3.91319000	0.04592300	-0.09635700
C	2.29364700	-1.05987000	0.24746400
H	1.89682900	0.98217700	0.09918800
H	1.94823100	-2.08896000	0.29827500
H	3.36873700	-0.91337700	0.28539400

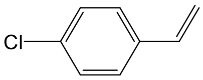

	x	y	z
C	-0.01018800	-0.04014000	0.08130700
C	1.45974700	-0.01451400	0.14068900
C	-0.70928700	1.17386000	-0.02467300
C	-0.76438100	-1.22709200	0.12523500
C	-2.15339600	-1.20474200	0.06625100
C	-2.10088800	1.21569700	-0.08499300
C	-2.81466400	0.02076300	-0.03881500
H	-0.26213000	-2.18628400	0.20670800
H	-2.72324200	-2.12728600	0.10104100
H	-0.15160700	2.10661100	-0.06067000
H	-2.62500800	2.16196600	-0.16669300
Cl	-4.57119100	0.05284500	-0.11365200
C	2.29091000	-1.05919500	0.24271800
H	1.89510300	0.98365400	0.09435600
H	1.94505400	-2.08805900	0.29491300
H	3.36605800	-0.91362400	0.27794900

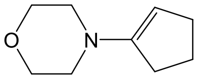

	x	y	z
O	0.01238700	0.07756400	-0.12144600
C	1.42323400	0.01129600	0.02801700
C	-0.42326800	1.42998200	-0.11067900
C	1.87294600	0.64924100	1.33989800
C	-0.04031900	2.13874600	1.18718400
N	1.39947300	2.03577900	1.40927500
H	-1.51106000	1.40775600	-0.22650600
H	1.68991200	-1.04976200	0.00761300
H	-0.60880000	1.70736100	2.03090800
H	1.48146400	0.05147500	2.18300600
H	0.01179300	1.97189700	-0.96683400
H	1.91804500	0.51824800	-0.81725700
H	-0.30610300	3.19846300	1.11133400
H	2.96512000	0.63457900	1.39643400
C	1.93910200	2.84962100	2.41028000
C	3.42131700	2.78378500	2.74196600
C	1.31494700	3.78135900	3.15873100
C	3.66134600	4.09453900	3.52701600
C	2.27150500	4.50329800	4.07799700

(continues)

	x	y	z
H	0.24947200	3.98296000	3.15572400
H	4.05001300	2.71040000	1.84560200
H	3.64491000	1.90035900	3.35869700
H	2.13641300	5.59330200	4.07603600
H	4.01671700	4.86648700	2.83499300
H	4.41861800	3.98506200	4.31012200
H	2.14066100	4.18213200	5.12419700

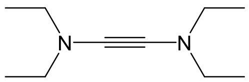

	x	y	z
C	2.15309300	3.67194700	3.78750800
N	2.33646200	3.19962700	5.02902400
C	1.93552800	4.07575100	2.65324500
N	1.70504600	4.46189200	1.38999300
C	1.16931700	2.71948300	5.78069100
C	3.57992100	3.53044000	5.73555300
C	2.14810500	3.59042000	0.29370200
C	1.39157200	5.87170200	1.12709200
H	0.64515500	1.99587400	5.14496500
H	1.54192200	2.17003500	6.65376300
C	0.18750600	3.81207100	6.21823000
H	0.64472800	4.50646100	6.93133200
H	-0.14732200	4.38618800	5.34761000
H	-0.69426200	3.36657300	6.69488300
H	3.66793300	2.84718000	6.58916600
H	4.41066600	3.30460500	5.05664700
C	3.69192800	4.98488900	6.20665800
H	3.56307400	5.66631500	5.35870000
H	2.93309700	5.22710400	6.95847900
H	4.67873700	5.17033200	6.64839500
H	1.75415400	2.58681200	0.49365300
H	1.66863800	3.94683400	-0.62614200
C	3.66700900	3.50893700	0.10528800
H	4.09164400	4.47466600	-0.18934200
H	4.14486000	3.19519000	1.03959500
H	3.91816800	2.77597800	-0.67117200
H	0.97703700	5.93544600	0.11354500
H	0.59291500	6.16315400	1.81945600
C	2.57143000	6.83839900	1.27950800
H	3.00924900	6.74320300	2.27911600
H	3.35639000	6.63953100	0.54195200
H	2.23754700	7.87511400	1.14895700
